# Population-Based Assessment of Hypertension Epidemiology and Risk Factors among HIV-Positive and General Populations in Rural Uganda

**DOI:** 10.1371/journal.pone.0156309

**Published:** 2016-05-27

**Authors:** Dalsone Kwarisiima, Laura Balzer, David Heller, Prashant Kotwani, Gabriel Chamie, Tamara Clark, James Ayieko, Florence Mwangwa, Vivek Jain, Dathan Byonanebye, Maya Petersen, Diane Havlir, Moses R. Kamya

**Affiliations:** 1 Infectious Diseases Research Collaboration, Kampala, Uganda; 2 Harvard University, Boston, Massachusetts, United States of America; 3 University of California, San Francisco, San Francisco, California, United States of America; 4 Kenya Medical Research Institute (KEMRI), Nairobi, Kenya; 5 University of California, Berkeley, California, United States of America; 6 Makerere University College of Health Sciences, Kampala, Uganda; British Columbia Centre for Excellence in HIV/AIDS, CANADA

## Abstract

**Background:**

Antiretroviral therapy scale-up in Sub-Saharan Africa has created a growing, aging HIV-positive population at risk for non-communicable diseases such as hypertension. However, the prevalence and risk factors for hypertension in this population remain incompletely understood.

**Methods:**

We measured blood pressure and collected demographic data on over 65,000 adults attending multi-disease community health campaigns in 20 rural Ugandan communities (SEARCH Study: NCT01864603). Our objectives were to determine (i) whether HIV is an independent risk factor for hypertension, and (ii) awareness and control of hypertension in HIV-positive adults and the overall population.

**Results:**

Hypertension prevalence was 14% overall, and 11% among HIV-positive individuals. 79% of patients were previously undiagnosed, 85% were not taking medication, and 50% of patients on medication had uncontrolled blood pressure. Multivariate predictors of hypertension included older age, male gender, higher BMI, lack of education, alcohol use, and residence in Eastern Uganda. HIV-negative status was independently associated with higher odds of hypertension (OR 1.2, 95% CI: 1.1–1.4). Viral suppression of HIV did not significantly predict hypertension among HIV-positives.

**Significance:**

The burden of hypertension is substantial and inadequately controlled, both in HIV-positive persons and overall. Universal HIV screening programs could provide counseling, testing, and treatment for hypertension in Sub-Saharan Africa.

## Introduction

Hypertension (HTN) is the leading risk factor for cardiovascular and cerebrovascular mortality worldwide [1], and is thought to be responsible for 45% of deaths due to heart disease and 51% of deaths due to stroke in 2013 [2,3]. The age-adjusted adult prevalence of HTN in Sub-Saharan Africa is the highest in the world at approximately 46% [3]. The World Health Organization (WHO) projects that non-communicable diseases (NCDs), such as HTN, will cause 50% of total mortality in Sub-Saharan Africa in 2015, and 61% by 2030 [4,5]. Although risk factors for HTN are well-characterized in developed countries [3], they remain incompletely understood in Sub-Saharan Africa [6]. One study from Ethiopia reported older age, male sex, family history, physical inactivity, salt-rich diet, and obesity as HTN risk factors [7]. Similar results were recently reported in Uganda, but with a limited sample size (N < 4000) [8,9].

The HIV epidemic in Sub-Saharan Africa has further complicated efforts to both understand and control the HTN burden in the region. HIV is also more prevalent in Sub-Saharan Africa than anywhere else in the world [10]. Rapid scale-up of anti-retroviral therapy (ART) has resulted in declines in HIV-related morbidity and mortality [10–12]. With this success has come an aging population of individuals living with HIV who are susceptible to NCDs such as HTN [13]. Moreover, whether ART medications or HIV itself interact with traditional risk factors for HTN remains unclear [14]. One study in Italy reported higher HTN prevalence in HIV-positive compared to HIV-negative adults [15]; others, also in developed countries, reported no significant association between HIV status and HTN [14,16,17]. Three studies in Sub-Saharan Africa found HIV infection to be associated with lower prevalence of HTN [8,18,19], but others reported HIV infection to be positively associated with cardiovascular disease as a whole [20–22]. Thus far, data on the HIV-HTN relationship have been largely clinic-based, and often lacked HIV-negative comparison groups [13].

In this study, we sought to characterize the prevalence and awareness of HTN among HIV-positive persons and the general population, in a large population-based sample from rural Uganda communities. We also sought to determine risk factors for HTN in both populations.

## Materials and Methods

### Study Setting and Background

We studied 20 non-adjacent rural Ugandan communities (ten in Western Uganda and ten in Eastern Uganda) enrolled in the Sustainable East Africa Research in Community Health (SEARCH) Study (NCT01864603) [23]. Each community has 16–25 villages and approximately 10,000 persons (~50% adults age ≥18 years). As described previously [24–28], each SEARCH study community held a census to enumerate households and obtain consent from household heads, followed by a community health campaign (CHC) offering multi-disease screening, treatment and linkage to care. Each adult (aged ≥18) participant was offered point-of-care screening for HTN, HIV, and diabetes; persons screening positive for any condition were linked to care at a nearby health center. We measured participants’ height (to nearest 0.1 cm), and weight (to nearest kilogram). We also collected demographic and behavioral information, including age, gender, occupation, marital status, education level, and alcohol use.

### Measurement of Blood Pressure, Blood Glucose and HIV Serostatus

We measured blood pressure (BP) on all adults using electronic sphygmomanometers. Participants with an elevated initial pressure (systolic BP ≥140 mmHg or diastolic BP ≥ 90 mmHg) had two repeat measurements ≥1 minute apart. Additionally, all adult participants were asked whether they were aware of a prior diagnosis of HTN and, if so, whether they had taken anti-hypertensive medications (prescribed at a health facility) in the past three months. Each adult participant also underwent a finger-prick point-of-care random blood glucose test (Optium-Abbott), with diabetes defined as a random blood sugar >11 mmol/L (196 mg/dL) and pre-diabetes defined as a random blood sugar >7 mmol/L (126/ mg/dL) but ≤ 11 mmol/L. HIV rapid testing was performed and interpreted according to the Uganda national algorithm [29]. HIV-positive participants had fingerprick blood drawn for viral load measurement using previously described methods [30]. All HIV-positive persons, regardless of prior knowledge of HIV status or ART use, were offered a first-line ART regimen of efavirenz with emtricitabine and tenofovir disoproxil fumarate at their local health center, as detailed in the SEARCH study protocol [23].

### Classification of HTN and risk factors

We defined HTN as a systolic BP≥140 or diastolic BP≥90 mm Hg on all three measurements as per World Health Organization (WHO) guidelines [31], or self-reported current use of anti-hypertensives. Stage 1 HTN was defined as highest systolic BP <160 mmHg and highest diastolic BP <100 mmHg; other hypertensive participants were defined as stage 2 [32]. We used body mass index (BMI) to classify participants as underweight or normal weight (< 25 kg/m^2^), overweight (25.0–29.9 kg/m^2^), or obese (≥ 30 kg/m^2^). We classified alcohol use (yes/no) based on self-report of current intake. We classified marital status as ‘single’ for persons reporting no current/prior marriage, and ‘non-single’ otherwise (e.g., currently married, divorced, or widowed). We calculated a household wealth index using principal components analysis [33], based on ownership of livestock (cows, goats, and poultry) and household items (clock, radio, television, phone, refrigerator, bicycle, motorcycle, and electricity).

### Estimation of Prevalence and Predictors of Hypertension

All CHC participants aged ≥ 18 years and resident to the 20 selected communities were included in the analysis. We computed the prevalence of HTN both within the population sample, and normalized to the WHO standard population distribution [34]. We used logistic regression, adjusting for demographics, BMI, education, socioeconomic status, alcohol use, HIV status and viral suppression (for HIV+), to identify independent predictors of HTN, both overall and among HIV-positive persons. We excluded observations with missing data and accounted for clustering by household. R(version 3.1.2) was used for analysis [35].

### Ethics

Heads of household provided written consent for all household members. Additionally, we obtained verbal consent from all adults and verbal parental consent for all children below 18 years of age. We waived a requirement for written consent for all participants because CHC activities involve no more than minimal harm to participants and involve no procedures for which written consent would otherwise be required, and because of limited literacy in our study population. Instead, we read the consent script orally to all participants, obtained verbal agreement that participants understood the study, and answered any questions. We documented each participant’s consent using a unique biometric fingerprint, which we recorded electronically and stored securely. The study was approved by the Makerere University School of Medicine Research and Ethics committee; the Uganda National Council on Science and Technology; and the University of California, San Francisco Committee on Human Research. Each of these committees expressly reviewed and approved the waiver of written consent above.

## Results

### Participant Demographics

Across 20 Ugandan communities, 94,274 stable adult residents were enumerated: persons present in their current household for at least 6 months of the year. Of these residents, 65,544 (70%) attended a CHC and received screening for both HTN and HIV (Table 1). Overall, 59% of participants were female; the median age was 34, with an interquartile range of 24–48. 84% of participants were non-single, and 78% had no secondary education (including 71% of men and 83% of women). More men than women (38% vs. 12%) reported alcohol use. Overweight and obesity were higher in females (18% and 5%, respectively) than males (8% and 1%, respectively). Diabetes prevalence was low among both genders (1%). HIV prevalence was 5% overall, and was slightly higher in females (6%) than males (5%).

**Table 1:** Participant Demographics and Hypertension Prevalence, Treatment and Control.

	Overall N = 65,544, n(%)	Women N = 38,424, n(%)	Men N = 27,120, n(%)
**Region**			
Eastern Uganda	33,342 (51%)	19,294 (50%)	14,048 (52%)
Western Uganda	32,202 (49%)	19,130 (50%)	13,072 (48%)
**Age (years)**			
18–29	25,360 (39%)	14,912 (39%)	10,448 (39%)
30–44	20,570 (31%)	12,296 (32%)	8,274 (31%)
45–59	11,599 (18%)	6,655 (17%)	4,944 (18%)
≥60	8,015 (12%)	4,561 (12%)	3,454 (13%)
**Marital status**^a^			
Single	10,344 (16%)	3,896 (10%)	6,448 (24%)
Non-Single (Married, Divorced; Widowed)	55,108 (84%)	34,486 (90%)	20,622 (76%)
**Education level**^b^			
No education	13,043 (20%)	9,869 (26%)	3,174 (12%)
Primary or less	37,933 (58%)	21,813 (57%)	16,120 (60%)
Any secondary or beyond	14,492 (22%)	6,710 (17%)	7,782 (29%)
**Alcohol use**^c^			
No	50,843 (78%)	33,956 (88%)	16,887 (62%)
Yes	14,679 (22%)	4,460 (12%)	10,219 (38%)
**BMI (kg/m**^**2**^**)**^d^			
<18 (underweight)	10,112 (16%)	4,634 (13%)	5,478 (21%)
≥18-<25 (normal)	41,926 (67%)	23,786 (65%)	18,140 (70%)
≥25-<30 (overweight)	8,622 (14%)	6,489 (18%)	2,133 (8%)
≥30 (obese)	2,038 (3%)	1,751 (5%)	287 (1%)
**Diabetes**^e^			
Pre-Diabetes	7,303 (11%)	4,228 (11%)	3,075 (11%)
Diabetes	521 (1%)	285 (1%)	236 (1%)
**HIV+**^f^	3,545 (5%)	2,304 (6%)	1,241 (5%)
**Hypertension**^g^ (of which:)	8,650 (14%)	4,734 (13%)	3,916 (15%)
Hypertensive by BP	8,021 (13%)	4,247 (12%)	3,774 (15%)
Stage 1	2,718 (34%)	1,240 (29%)	1,478 (39%)
Stage 2	5,303 (66%)	3,007 (71%)	2,296 (61%)
Hypertensive by history	629 (1%)	487 (1%)	142 (1%)
Aware of hypertension	1,797 (21%)	1,345 (28%)	452 (12%)
Receiving medication	1,260 (15%)	949 (20%)	311 (8%)
BP controlled	629 (50%)	487 (51%)	142 (46%)
BP uncontrolled	631 (50%)	462 (49%)	169 (54%)

Pre-Diabetes: Random blood sugar >7 mmol/L but ≤ 11 mmol/L; Diabetes: Random blood sugar >11 mmol/L; BP: blood pressure; Hypertensive by BP: Systolic BP≥140 mmHg or Diastolic BP ≥90 mmHg on 3/3 readings; Hypertensive by history: self-reported hypertensive, on anti-hypertensive medications and negative by BP criteria.

^a^N = 65452 (missing = 92), women = 38382, men = 27070

^b^N = 65468 (missing = 76), women = 38392, men = 27076

^c^N = 65522 (missing = 22), women = 38416, men = 27106

^d^N = 62698 (missing = 2846), women = 36660, men = 26038

^e^N = 64501 (missing = 1043), women = 37760, men = 26741

^f^N = 64736 (missing = 808), women = 37974, men = 26762

^g^N = 61880 (missing = 3664), women = 36210, men = 25670

### Hypertension prevalence, awareness, treatment and control

Overall, 8,650 participants had HTN, representing 14% prevalence (women: 13%; men: 15%; Table 1). Normalized to WHO world standard population distribution [34], HTN prevalence was 17% overall (women: 16%; men: 17%). In total, 8,021/ 8,650 participants (93%) met HTN criteria based on CHC BP screening while 629/8,650 (7%) reported a previous HTN diagnosis and current HTN medication use but had a negative HTN screen. Among the 8,021 participants with a positive HTN screen, 66% had Stage 2 HTN. Only 21% of hypertensive adults were aware of their diagnosis (women: 28%; men: 12%). Of those aware of their HTN, only 15% reported taking anti-hypertensive medication, and of persons taking medications, only 50% were normotensive (Table 1).

### Predictors of Hypertension in the Overall Population

Risk factors for HTN included Eastern region residence, male gender, older age, no education (as compared to primary education), alcohol use, and higher BMI (Table 2). However, the odds of HTN were 1.2-fold higher among HIV negative persons compared to HIV positive persons (95% CI: 1.1–1.4).

**Table 2:** Predictors of Hypertension in multivariable analysis.

	Among all Adults^a^		Among HIV+ Adults^b^	
Variable	Adj. Odds Ratio (95%CI)	p-value	Adj. Odds Ratio (95%CI)	p-value
**Region**				
Western Uganda (ref)	-	-	-	-
Eastern Uganda	1.26 (1.2, 1.3)	<0.0001	1.34 (1.0, 1.7)	0.027
**Sex**				
Female (ref)	-	-	-	-
Male	1.23 (1.2, 1.3)	<0.0001	1.20 (0.9, 1.6)	0.23
**Age**				
18–29 (ref)	-	-	-	-
30–44	2.27 (2.1, 2.5)	<0.0001	2.21 (1.4, 3.6)	0.0015
45–59	5.58 (5.1, 6.1)	<0.0001	4.65 (2.8, 7.7)	<0.0001
60+	12.66 (11.6, 13.9)	<0.0001	11.82 (6.6, 21.3)	<0.0001
**Education Level**				
Less than primary (ref)	-	-	-	-
Primary	0.86 (0.8, 0.9)	<0.0001	0.70 (0.5, 1.0)	0.024
More than primary	0.96 (0.9, 1.1)	0.40	0.85 (0.6, 1.2)	0.41
**Socio-economic Status**				
1^st^ Wealth quintile (ref)	-	-	-	-
2^nd^ Wealth quintile	0.96 (0.9, 1.1)	0.40	1.09 (0.7, 1.7)	0.69
3^rd^ wealth quintile	0.99 (0.9, 1.1)	0.84	1.30 (0.9, 2.0)	0.22
4^th^ wealth quintile	1.02 (0.9, 1.1)	0.65	1.64 (1.1, 2.4)	0.013
5^th^ wealth quintile	0.99 (0.9, 1.1)	0.83	1.68 (1.1, 2.5)	0.013
**Alcohol Use**				
No (ref)	-	-	-	-
Yes	1.30 (1.2, 1.4)	<0.0001	1.09 (0.8, 1.5)	0.57
**BMI (kg/m**^**2**^**)**				
Normal Weight or Underweight (ref)	-	-	-	-
Overweight	1.90 (1.8, 2.0)	<0.0001	1.55 (1.1, 2.2)	0.016
Obese	2.99 (2.7, 3.4)	<0.0001	2.91 (1.6, 5.3)	0.0004
**HIV status**				
Negative (ref)	-	-	-	-
Positive	0.82 (0.7, 0.9)	0.0022		
**Viral suppression (if HIV+, viral load ≤ 500 copies/mL)**				
No (ref)	-	-	-	-
Yes	-	-	1.07 (0.8, 1.4)	0.62

^a^Analysis restricted to all adults without missing data (N = 54,598)

^b^Analysis restricted to all HIV+ adults without missing data (N = 2,599)

### Prevalence and Predictors of Hypertension in the HIV+ Population

The prevalence of HTN was greater among HIV-negative adults (14%) than among HIV-positive adults (11%). Among HIV-positive adults with HTN, 20% reported prior knowledge of HTN, and 14% reported taking medication. Similar to the overall population, 46% of HIV-positive adults with HTN achieved BP control with anti-hypertensive medications. After multivariate adjustment, significant predictors of HTN among persons with HIV were Eastern region residence; older age; no education (as compared to primary education); higher wealth index (income in top two quintiles); and higher BMI. Viral suppression of HIV did not significantly predict HTN (adjusted OR 1.1; 95% CI: 0.8–1.4).

## Discussion

We report a 14% overall adult prevalence of HTN in a large Ugandan population sample. We also found low awareness of HTN (21%), infrequent use of anti-hypertensive medication (15%), and suboptimal levels of blood pressure control in persons on medication (50% of users; 7% of all persons with HTN). In the HIV-positive population, results were similar: HIV prevalence was 11%, only 20% of adults were aware of their diagnosis, 14% were on treatment, and 46% were of these normotensive (6% of all persons with HTN). These stark results are consistent with other studies in Ugandan populations [8,9,25–27,36–39], and other Sub-Saharan African populations [19,40–46], that have reported a HTN prevalence of 15–50%.

We found substantial differences in HTN control by gender. HTN awareness was higher in women than men (28% vs. 12%), consistent with other Ugandan data [26,36]. This result may be due to women more frequently engaging with health services because of their maternal and child care roles [43]. Adjusted for age according to the WHO world standard population, the overall prevalence of HTN is 17%, which is lower than some recent studies [9], but comparable to rates in the United Kingdom (18.6%) [46], and other developed countries [47–49]. These results suggest a large and unaddressed burden of HTN in Uganda that requires concerted screening and control efforts in both HIV-positive persons and the nation overall. Meeting this need requires health system strengthening efforts such as community health campaigns or community outreach, an approach validated in the control of asymptomatic diseases such as HIV and HTN [28,50,51].

We report HTN risk factors that parallel prior Ugandan findings [8,9,38], as well as data from developed nations [3,47,52]. Risk factors include male gender and older age, as well as modifiable factors such as overweight and obesity; alcohol consumption; having no primary education; and residence in Eastern Uganda. All six of these factors were predictive of HTN in the general population, but male gender and alcohol consumption were not predictive of HTN in persons with HIV. Conversely, high socioeconomic status (top two income quintiles) was associated with HTN in HIV-positive persons only. These discrepancies may reflect a difference in sample size (and hence statistical power) between the two groups, given that the association between alcohol use, male gender, and HTN risk is well-understood [3, 53, 54]. The association between Eastern Ugandan residence and HTN may be related to unmeasured confounders, such as differences in dietary salt intake between the two regions.

These results suggest an opportunity to employ HTN treatment and prevention programs in Uganda that have been successful in other countries, e.g., alcohol and salt limitation, and frequent exercise [3,53,54]. As Uganda’s economic development accelerates, greater primary education may increase HTN awareness and control. However, rising wealth indices may conversely create diet and exercise changes that could increase HTN rates. Thus, more research is needed on the impact of education and socio-economic status on HTN in Sub-Saharan Africa, both in the general population and among persons living with HIV.

Notably, HIV-positive status was associated with a lower risk of HTN (11% prevalence among HIV-negative persons, but 14% among HIV+ persons), consistent with other studies in Sub-Saharan Africa, and in Uganda specifically [8,18,19]. After controlling for other risk factors, the odds of HTN were 20% higher for HIV-negative persons than HIV-positive persons. This result eludes clear biological explanation. Antiretroviral medications (and HIV itself) may raise BP via physiologic changes such as glucocorticoid and insulin resistance [55,56]. Moreover, studies in developed countries have observed higher blood pressure in persons with HIV than HIV-negative controls [14,15].

Several possible explanations are plausible, and will need further study. First, SEARCH Study community health campaigns measure BP just prior to testing for HIV. HIV-positive persons who know their HIV status prior to testing may have lower anxiety (and thus BP). Biological mechanisms are also plausible. Genetic factors (e.g. CCR5 mutation) are protective against HIV infection [57]. Similarly, genetic factors (both predisposing and protective) have been linked to HTN [58]. It is thus possible that genetic factors predisposing to HIV infection might be concomitantly protective against HTN. Alternatively, the observed negative association between HIV and HTN may be due to a paucity of persons suffering from both conditions (due to survival bias attributable to mortality). A longitudinal study tracking populations positive for HTN alone; HIV alone; or the two conditions concurrently, could shed light on how these risk factors interact.

Among HIV-positive adults, we found no substantial association between HTN and viral suppression. While current evidence suggests that circulating viremia in HIV infection can cause inflammatory and endovascular changes that promote HTN [59], antiretroviral medications themselves may directly cause or precipitate HTN [17,56]. It is therefore possible that persons with HIV who achieve viral suppression through ART concurrently *decrease* risk of HTN (due to control of viremia) and *increase* of HTN via ART medications: two opposing effects that may offset one another. Future longitudinal studies could investigate this hypothesis by investigating HIV-positive persons on ART who fail to achieve viral suppression; or, conversely, HIV-positive persons who achieve viral suppression without ART (i.e., elite suppressors).

Our study has two important limitations. It was cross-sectional: we could not assess temporal relationships between risk factors and HTN outcomes. We also could not adjust for certain unmeasured potential confounders, including: physical activity, smoking, renal function, lipid levels and family history of HTN. However, our previous work suggests that smoking in this population is relatively uncommon (12–18%) [26,27], and that tobacco use is not significantly associated with HTN [26]. Moreover, our study did adjust for many potential confounders, such as age, gender, education level, socio-economic status, alcohol use, BMI, and region.

We report a large burden of HTN in Uganda, which remains largely undiagnosed and uncontrolled in both HIV-positive persons and the general population. The risk factors for HTN in both groups appear to be similar to those described in developed countries, such as older age, male gender, obesity, and alcohol use. Although we found HIV infection to be negatively associated with HTN after adjusting for common risk factors, the burden of hypertensive disease in persons with HIV remains large and inadequately controlled. These results suggest that programs for HTN screening and control are urgently needed in Uganda. Increasing access to ART for HIV in Sub-Saharan Africa, along with improved socioeconomic opportunity and an aging population, will likely only increase the burden of HTN and other NCDs in the region. However, programs for universal screening and treatment of HIV offer a unique opportunity to screen for and educate on HTN in a population with minimal prior access to care [25–27]. Health systems in Sub-Saharan Africa could adapt these models as they work to respond to a growing NCD burden, while concurrently ensuring universal access to HIV screening and care.
